# The impact of COVID-19 vaccine distribution channels on equity-deserving populations: a Canadian population-based cohort study using administrative data

**DOI:** 10.1186/s12889-025-24824-4

**Published:** 2026-01-09

**Authors:** Alan Katz, Kris Aubrey-Bassler, Fariba Aghajafari, Gillian Fransoo, Noah M Ivers, Jeffrey C Kwong, Rahim Moineddin, Lena Nguyen, Danielle Saj, Carole Taylor, Ross E G Upshur, Jennifer Woodrow, Sabrina T Wong, Hui Xiong, Monica Aggarwal

**Affiliations:** 1 Manitoba Centre for Health Policy, Max Rady College of Medicine, University of Manitoba, Room 408-727 McDermot Ave, Winnipeg, MB R3E 3P5 Canada; 2 Department of Family Medicine, Max Rady College of Medicine, University of Manitoba, S100, 750 Bannatyne Ave, Winnipeg, MB R3E 0W2 Canada; 3 Primary Healthcare Research Unit, Faculty of Medicine, Memorial University, 300 Prince Philip Dr, St. John’s, NL A1B 3V6 Canada; 4 Discipline of Family Medicine, Faculty of Medicine, Memorial University, 300 Prince Philip Dr, St. John’s, NL A1B 3V6 Canada; 5 Department of Family Medicine, Cumming School of Medicine, University of Calgary, Sunridge Family Medicine Teaching Centre, 2685 - 36 Street NE, Calgary, AB T1Y 5S3 Canada; 6 Department of Community Health Sciences, Cumming School of Medicine, University of Calgary, Sunridge Family Medicine Teaching Centre, 2685 - 36 Street NE, Calgary, AB T1Y 5S3 Canada; 7 Department of Family and Community Medicine, Women’s College Hospital – University of Toronto, 77 Grenville St, Toronto, ON M5S 1B3 Canada; 8 Department of Family and Community Medicine, University of Toronto, 500 University Ave, 5th floor, Toronto, ON M5G 1V7 Canada; 9 Institute for Clinical Evaluative Sciences (ICES) Central, 122-2075 Bayview Ave, Toronto, ON M4N 3M5 Canada; 10 Dalla Lana School of Public Health, University of Toronto, 155 College St, 6th floor, Toronto, ON M5T 3M7 Canada; 11 Data and Information Services, Newfoundland and Labrador Health Services, 70 O’Leary Ave, St. John’s, NL A1B 2C7 Canada; 12 School of Nursing, Faculty of Applied Sciences, University of British Columbia, Y201-2211 Wesbrook Mall, Vancouver, BC V6T 2B5 Canada; 13 Centre for Health Services and Policy Research, University of British Columbia, 2206 East Mall, Vancouver, BC V6T 1Z3 Canada

**Keywords:** SARS-CoV-2, Routinely collected health data, Vaccines, Public health, Health policy, Health equity

## Abstract

**Background:**

Uptake of COVID-19 vaccines among equity-deserving populations was a challenge throughout the Canadian vaccination campaign. This necessitated the implementation of vaccine distribution strategies that targeted specific populations. As a result, different vaccine channels were introduced in each province. The purpose of this study is to examine the effect of COVID-19 vaccine distribution channels on short-term vaccination rates amongst equity-deserving populations.

**Methods:**

Retrospective, population-based cohort using linked administrative claims data based in the Canadian provinces of Manitoba, Ontario, and Newfoundland and Labrador. The study population included all residents, 12 years and older, who were living in one of the three provinces with at least 1 day of health coverage between Jan 1, 2021, and Dec 31, 2021. The index event was receiving one COVID-19 vaccination. Difference-in-differences analyses were used to evaluate the impact of various COVID-19 vaccine distribution channels, such as directed at-risk, mass vaccination clinics, pharmacies, primary care offices, and long-term care facilities, on the gap in vaccination coverage between select equity-deserving and non-equity deserving populations. The equity-deserving populations included having lower household income, identifying as a visible minority, being a recent immigrant, and being an adult without a high school diploma.

**Results:**

The total COVID-19-vaccinated population as of December 31, 2021 was 998,906 (85% of total population) in Manitoba, 10,866,548 (89% of total population) in Ontario, and 485,901 (99% of total population) in Newfoundland and Labrador. The analyses were limited by significant data challenges; data on where (which channel) specific individuals received their vaccination were limited. Though findings were mixed, in general, the COVID-19 vaccine coverage one month after the initiation of a new vaccine distribution channel increased less for the equity deserving groups than those of the general population.

**Conclusion:**

An increase in the observed equity gaps immediately after new vaccine channel introductions may be explained by new channels providing vaccines at a lower volume than mass-vaccination clinics or may reflect limitations in the data infrastructure. The collection of uniform data across Canada should be prioritized to facilitate comprehensive evaluation of the health system response to future pandemics, including the ability to monitor the impact of the response on population inequities.

**Supplementary Information:**

The online version contains supplementary material available at 10.1186/s12889-025-24824-4.

## Key Findings

### What was studied

Whether the introduction of new COVID-19 vaccine distribution channels increased vaccine uptake among equity-deserving populations in three Canadian provinces.

### Why it matters

Learning from past health crises can guide future system planning and implementation. Understanding how vaccine distribution strategies affected marginalized populations is key to building more equitable public health systems.

### What we found

- New channels were not associated with significant increases in uptake among equity-deserving populations compared to the general population.
- Uptake increased less in geographical areas with high proportions of visible minority adults, recent immigrants, and individuals without a high school diploma.

### What it means

Vaccine uptake is complex and dependent on multiple factors. Observed disparities may reflect differences in the delivery capacity of vaccine channels or limitations in available data. Improved data systems are needed to better assess and address inequities.

### Recommendations

Prioritize consistent, high-quality data collection across jurisdictions to monitor and improve equity in future public health responses.

## Background

In March 2020, the World Health Organization characterized COVID-19 as a pandemic [1]. Since then, COVID-19 has caused significant societal disruptions worldwide, impacting individuals mentally, socially, and physically [2, 3]. COVID-19 vaccines were deployed to reduce the burden of acute infection, as well as related long-term complications, mortality, and hospitalizations [4–8]. Despite recommendations by government and public health authorities to get vaccinated, uptake among equity-deserving communities (e.g. racialized peoples (a person or group of people categorized by ethnic or racial characteristics and subjected to discrimination), immigrants, people experiencing homelessness, lower socioeconomic status) was a challenge in Canada throughout the initial vaccination campaigns [9–15]. While vaccine hesitancy and the widespread circulation of misinformation certainly contributed to lower vaccination coverage among some of these equity-deserving populations, difficulties accessing the vaccine were also a significant barrier [12, 16–18]. Vaccine access was impacted by factors such as linguistic or literary limitations, inflexible work schedules, inaccessible scheduling systems, unreliable transportation options, and culturally unsafe vaccine sites [13, 16]. 

The challenge of healthcare-related inequities among society’s less privileged populations is not unique to the COVID-19 pandemic. The Theory of Fundamental Cause suggests that health-related disparities persist among marginalized populations due to a lack of resources such as money, knowledge, prestige, power, and beneficial societal connections [19, 20]. The consequences are that new knowledge and medical developments related to disease control will favor less marginalized populations who have access to abundant resources [19]. As a result, medical progress sometimes exacerbates health inequities [19]. This highlights the need for innovative vaccine distribution strategies that specifically target populations facing barriers to being vaccinated.

While Canada’s federal government oversees health policies related to international travel, vaccine approval, procurement, and distribution to provinces, it is the provincial and territorial governments that manage vaccine prioritization, administration, monitoring, and coverage reporting [21, 22]. This resulted in differing vaccine distribution approaches across jurisdictions which was previously described in our protocol paper [22, 23]. As outlined in that paper, the federal government allocated doses to provinces on a per capita basis, while provincial and territorial governments determined how and where vaccines were delivered within their own jurisdictions [22]. These distribution decisions reflected the local infrastructure, population needs, equity goals, and access challenges unique to each province or territory.

The rollout of vaccines began on December 15th, 2020, in the province of Ontario and the following day in Manitoba and Newfoundland and Labrador [24–26]. The first vaccinations in Ontario and Manitoba were administered in hospital settings [27, 28]. Initially vaccine supplies were limited, and eligibility, determined by each province, changed as vaccine supplies increased. The initial provincial vaccine rollout was impacted by vaccine supply shortages and transportation challenges [27–29]. In the early stages of the campaign, vaccines were shipped directly to vaccination sites due to concerns related to product stability and cold chain requirements [28]. These factors led to the implementation of mass vaccination sites, known as “supersites” in some provinces, as the primary vaccine delivery channel [29, 30]. Supersites enabled rapid, large-scale vaccination and were adaptable to evolving vaccine supply [31]. This centralized model also facilitated better control over storage and handling, especially for mRNA vaccines requiring ultra-cold conditions. As evidence on vaccine stability evolved, the provinces expanded delivery models, enhancing reach and flexibility in later phases [32]. 

While supersites were successful at vaccinating many people, they failed to mitigate socioeconomic inequities and counter the vulnerabilities of equity-deserving groups [23]. Access barriers, such as a need for technology to book appointments, time off from work, and transportation, as well as social barriers such as structural racism, distrust in the healthcare system, and a lack of cultural safety at vaccination sites were not considered in the initial rollout of vaccines from supersites [23]. This highlighted the necessity for alternative vaccine delivery channels that targeted equity-deserving populations. Manitoba, Ontario, and Newfoundland and Labrador utilized different alternative channels influenced, in part, by advocacy by different community organizations, previous experiences with the H1N1 influenza pandemic, and guidance of public health leadership in each jurisdiction (Table 1) [33–38]. In particular, the severe impact of H1N1 on First Nations communities informed their prioritization during the COVID-19 rollout, leading to the implementation of Indigenous-led strategies and early access initiatives [39]. A pending policy review by Aggarwal et al. describes these initiatives in greater detail. [personal communication Aggarwal M, May 2025]

**Table 1:** Vaccine distribution channels utilized in Manitoba, Ontario, and Newfoundland and Labrador (Newfoundland)

Date	Vaccine Channel	Notes
Manitoba		
Dec 2020	Hospitals	Served as pilot sites that informed the future phases of the rollout. [28]
Jan 2021	Mass vaccination clinics	10 clinics across the province served as distribution points for focused immunization teams pop-up clinics. [31]
Jan 2021	Focused immunization teams (FITs)	FITs targeted personal care home residents and congregate living sites. [28, 31, 40]
Feb 2021	Pop-up clinics	Targeted rural and northern areas of the province. [28, 31, 40]
March 2021	Medical offices and pharmacies	Capitalized on existing patient-provider relationships and partnerships. [28, 31]
May 2021	Urban indigenous clinics	An Indigenous vaccine committee provided culturally safe support for indigenous populations and people experiencing homelessness. [41, 42]
Ontario		
Dec 2020	Hospitals	Served as pilot sites that informed future phases of the rollout. [38, 43]
Jan 2021	Mass vaccination clinics	
Jan 2021	Mobile immunization teams and pop-up clinics	Targeted long-term care homes, Indigenous peoples and other rural or materially/socially deprived populations. [33, 44, 45]
March 2021	Medical offices and pharmacies	
April 2021	Black client-centered clinics	326 total clinics initiated by the Black Physicians Association of Ontario in partnership with the Public Health Agency of Canda. [46, 47]
April 2021	Hot-spot distribution	Targeted designated COVID-19 hotspots via mobile teams, workplace clinics, community-based pop-up clinics, and site-specific mass vaccination clinics. [33, 48–51]
Newfoundland and Labrador		
Dec 2021	Mass vaccination clinics	
March 2021	Mobile clinics	Targeted smaller communities and homebound individuals. [29, 30]
May 2021	Community public health vaccine clinics	Partnered with large businesses, industries, and community-based settings. [29, 30, 52]
June 2021	Primary care physician offices and pharmacies	
Jan 2022	Walk-in vaccine clinics	Outside of the health authority, staffed by medical students, medical residents, nurses, and pharmacists. [53]

Communication strategies in Ontario, Manitoba, and Newfoundland also evolved over time to better reach equity-deserving populations. Provinces introduced multilingual materials and partnered with community organizations and ethnic media to deliver culturally relevant messaging [54–56]. These approaches aligned with evidence that newcomer and racialized communities often relied on trusted local networks for COVID-19 information.

There remains a paucity of literature on how different distribution approaches impacted uptake and acceptance of COVID-19 vaccines among equity-deserving populations in Canada, nor are there best-practice guidelines on effective distribution strategies for equity-deserving populations [22, 57]. Some international studies have examined vaccine delivery approaches, including targeted outreach in the United States [58]. In addition, modelling studies have explored logistical challenges such as cold chain requirements, the impact of vaccine uptake on transmission, and disruptions to routine immunization [59, 60]. One study also compares vaccine distribution challenges in African countries and the United States. However, few of these studies focus specifically on equity-related outcomes [61]. Therefore, in this study we sought to assess whether the addition of new vaccine channels was associated with greater vaccine uptake, particularly for equity-deserving.

## Methods

### Study design and setting

A quasi-experimental study design, using administrative data from the Canadian provinces of Manitoba, Ontario, and Newfoundland, was implemented to examine the effect of vaccine distribution channels amongst equity-deserving populations and to evaluate the impact of vaccine distribution channels on equitable vaccination rates. The equity-deserving populations included having lower household income, identifying as a visible minority (persons, other than indigenous people, who are non-Caucasian in race or non-white in colour), being a recent immigrant, and being an adult without a high school diploma [62]. These populations were selected based on previous literature as well as data availability in the three jurisdictions [63–71]. 

### Data sources

We focused on Manitoba, Ontario, and Newfoundland due to the availability of both vaccine channel data and linked population-level vaccination rates [72–74]. We initially aimed to also include British Columbia, Alberta, Quebec, and Nova Scotia in our analyses; however, these provinces were unable to provide the necessary data. The selection of specific equity-deserving groups was also directed by data availability. The specific databases used in the three jurisdictions are listed in Additional File 1. Access to the database populations was not limited. All records in the study datasets were de-identified (name and addresses removed) and linked at the individual level using unique study identifiers.

### Study population and time period

We began with all residents of each province who had at least 1 day of valid provincial health coverage prior to January 1, 2021, and were living within that province with at least 1 day of health coverage between January 1, 2021, and December 31, 2021. We chose this study period because it covered the timeframe when new channels were introduced and was prior to the widespread circulation of the Omicron variant. The index event was receiving a COVID-19 vaccination. We excluded residents for whom we did not have a valid postal code, who had less than one day of coverage before the index date, who were living outside the province during the study period, or who were less than 12 years of age.

### Variables

The selection of equity-deserving groups for this study was informed by data availability as well as a literature review to identify demographic factors associated with low vaccine uptake [72–74]. We considered the proportion of the population aged 25–64 without a high school diploma as well as the proportion of the population who identify as a visible minority which are indicators of situational vulnerability and ethno-cultural composition, respectively, in the Canadian Index of Multiple Deprivation [75]. We also included household income quintile. Education, visible minority, and income were assigned at the neighbourhood level by ranking postal codes for the respective variable and then dividing the ranked postal codes into five equally sized quintiles. A value of 1 corresponds to neighbourhoods that were the least deprived, and a value of 5 corresponds to neighbourhoods that were the most deprived [75, 76]. Finally, we considered immigration status. Everyone born in Canada as well as any immigrant who has lived in Canada for more than 15 years was classified as a ‘non-immigrant’. Everyone who immigrated to Canada within the last 15 years was classified as an ‘immigrant’.

Verifiable vaccine channel data was not available at the level presented in Table 1; therefore, we aggregated data from certain channels into broader distribution site categories. The different vaccine channels analyzed in the three jurisdictions are described in Table 2. In this analysis, individuals living in rural communities or congregate settings were not categorized as minorities unless they also belonged to one of the previously defined equity-deserving groups.

**Table 2:** Descriptions of the vaccine channels analyzed in Manitoba, Ontario, and Newfoundland

Vaccine Channel	Description
Manitoba	
Directed At-Risk	All vaccinations administered within First Nations Health Authorities on reserve
Mass Vaccination Clinics	All vaccinations administered within Manitoba Health Authorities
Pharmacy	All vaccinations administered in pharmacies
Medical Practitioner	All vaccinations administered in primary care/medical practitioner offices
Ontario^a^	
Congregate living	All vaccinations administered in long-term care, retirement home, assisted living, and other congregate care settings
Directed At-Risk	All vaccinations administered at/by mobile delivery sites, mobile team – homebound patients, occupational/workplace clinics, pop-up clinics, school-based clinics
Mass Vaccination Clinics	All vaccinations administered in mass vaccination, drive-though, or hospital-based clinics
Pharmacy	All vaccinations administered in pharmacies
Newfoundland and Labrador	
Electronic Medical Record (EMR)	All vaccination records that were entered into the EMR system including Public Health vaccination clinics, congregate living facilities (prisons, rotational work camps), schools, personal care homes, dialysis units, hospital inpatients, physician clinics, and home visits
Pharmacy	All vaccinations administered at community pharmacies
Long-Term Care	All vaccinations administered in long-term care facilities

^a^In Ontario, vaccines were also administered in primary care offices; however, we did not perform analyses on this data due to uncertainties about the accuracy of the channel classification

### Statistical analysis

Analyses of the linked administrative data were performed separately in each jurisdiction. We generated descriptive statistics, by province, to identify the count and proportion of individuals within equity-deserving and non-equity-deserving groups who received vaccinations. Then, for each jurisdiction, cumulative monthly COVID-19 vaccination proportions were calculated by dividing the number of individuals vaccinated in a specific month by the population eligible for vaccination in that month. Pre-post channel introduction comparisons were based on aggregate data using additive approaches and expressed as differences with 95% confidence intervals. Changes in the size of the gaps between equity-deserving populations and non-equity-deserving populations over time were estimated with the basic difference-in-differences design analyses to obtain absolute proportion differences with 95% confidence intervals. A basic difference-in-differences analysis is a quasi-experimental design that is used in observational settings to compare changes in outcomes over time between two populations adopting three key assumptions: (i) consistency, (ii) parallel trends, and (iii) no-anticipation assumptions [77]. The change in the gap was estimated by an interaction term between population groups (equity-deserving and non-equity-deserving) and time with a normal approximation for difference-in-differences of proportions. Aggregate results were compared across provinces.

This study was reported in accordance with the RECORD (REporting of studies Conducted using Observational Routinely-collected health Data) guidelines (Additional File 2). All statistical analyses were performed using SAS version 9.4 (SAS Institute, Cary, NC) software.

## Results

Table 3 presents the population characteristics for the three jurisdictions at the midpoint of the study period. While the age distribution varied, the patterns were very similar with Newfoundland having an older mean age (49.27 vs. 46.61 for Ontario and 44.99 for Manitoba). The sex and income quintile distributions did not differ. Newfoundland had a notably higher percentage of people living in postal codes with the lowest percentage of self-identified visible minorities (52.93 vs. 15.5 and 14.78% for Ontario and Manitoba) and the highest percentage of people without a high school diploma. Immigration data was not available for Newfoundland. The percentage of immigrants and non-immigrants living in Manitoba and Ontario were the same.

**Table 3:** Demographic characteristics of the total population (Jan 1, 2021 – Dec 31, 2021)

	Manitoba		Ontario		Newfoundland	
	*N*	%	*N*	%	*N*	%
Total population	1 179 399		12 155 147		486,879	
Age						
Age 12–17	102 012	8.7	776 776	6.4	32 134	6.6
Age 18–49	588 806	49.9	5 897 208	48.5	205 162	42.1
Age 50–59	173 410	14.7	2 003 775	16.5	85 259	17.5
Age 60–69	158 676	13.5	1 755 700	14.4	84 748	17.4
Age 70–79	99 167	8.4	1 123 352	9.2	56 192	11.5
Age 80+	57 328	4.9	589 336	4.9	23 384	4.8
Sex						
male	582 957	49.4	5 938 268	48.9	246 938	50.7
female	596 442	50.6	6 216 879	51.2	239 941	49.3
Income Quintile						
1 (lowest)	238 918	20.3	2 358 653	19.4	99 195	20.4
2	235 738	20.0	2 417 044	19.9	95 188	19.6
3	233 742	19.8	2 439 373	20.1	96 607	19.8
4	229 202	19.4	2 438 696	20.1	98 403	20.2
5 (highest)	232 273	19.7	2 473 803	20.4	96 033	19.7
Missing information	9 526	0.8	27 578	0.2	1 453	0.3
Proportion of people who self-identify as a visible minority^a^						
1 (lowest)	174 360	14.8	1 879 189	15.5	257 704	52.9
2	175 623	14.9	2 078 295	17.1		
3	234 022	19.8	2 178 865	17.9		
4	230 602	19.6	2 633 718	21.7	117 102	24.1
5 (highest)	264 743	22.5	3 337 555	27.5	110 191	22.6
Missing information	100 049	8.5	47 525	0.4	1 882	0.4
Proportion of people (25–64) without a diploma^a^						
1 (lowest)	234 246	19.9	2 188 348	18	127 586	26.2
2	246 432	20.9	2 898 874	23.8	111 722	23.0
3	215 512	18.3	2 526 145	20.8	97 738	20.1
4	203 865	17.3	2 371 493	19.5	80 814	16.6
5 (highest)	179 295	15.2	2 122 545	17.5	67 137	13.8
Missing information	100 049	8.5	47 742	0.4	1 882	0.4
Immigration Status^b^						
Immigrant (≤ 15 years)	114 890	9.7	1 144 951	9.4		
Non-immigrant (> 15 years)	1 064 509	90.3	11 010 196	90.6		

^a^Quintiles were derived from the Canadian Index of Multiple Deprivation by ranking postal codes and dividing them into five groups. For education, 1 = lowest and 5 = highest proportion without a high school diploma (least to most deprived). For visible minority, 1 = lowest and 5 = highest proportion of residents identifying as a visible minority

^b^Immigration status data was not available for Newfoundland and Labrador

The vaccination rates at the end of the study period are presented in Table 4. The rates in Newfoundland were the highest at over 99% for all population groups. In Ontario, 88.9% of males and 90.2% of females were vaccinated. Residents under 50 years old had slightly lower rates of vaccination than those over 50. The same age distribution was seen in Manitoba where the rates were generally lower than in Ontario (males 83.54 and females 85.82%).

Both Manitoba and Ontario demonstrated gradients in vaccination rates for each of the equity-deserving groups, with the lowest rates among those most vulnerable to health inequities. The overall rates in Newfoundland were too high to differentiate between groups. Vaccination rates over time for each equity deserving group are presented in Additional File 3.

**Table 4:** Demographic characteristics of the vaccinated population (Jan 1, 2021 – Dec 31, 2021)

	Manitoba		Ontario		Newfoundland	
	*N*	%	*N*	%	*N*	%
Total vaccinated	998 906	84.7	10 866 548	89.4	485 901	99.8
Age						
Age 12–17	84 368	82.7	687 652	88.5	32 074	99.8
Age 18–49	481 051	81.7	5 173 231	87.7	204 607	99.7
Age 50–59	151 072	87.1	1 819 321	90.8	85 114	99.8
Age 60–69	141 718	89.3	1 613 043	91.9	84 638	99.9
Age 70–79	90 155	90.9	1 044 074	92.9	56 128	99.9
Age 80+	50 542	88.2	549 227	91.8	23 340	99.8
Sex						
male	487 011	83.5	5 279 785	88.9	246 498	99.8
female	511 895	85.8	5 606 763	90.2	239 403	99.8
Income Quintile						
1 (lowest)	191 806	80.3	2 041 944	86.6	98 929	99.7
2	200 109	84.9	2 144 070	88.7	95 007	99.8
3	199 900	85.5	2 190 089	89.8	96 421	99.8
4	195 789	85.4	2 212 440	90.7	98 252	99.9
5 (highest)	202 907	87.4	2 274 774	92.0	95 843	99.8
Missing information	8 395	88.1	23 231	84.2	1 449	99.7
Proportion of people who self-identify as a visible minority^a^						
1 (lowest)	139 687	80.1	1 645 273	87.6	257 231	99.8
2	146 589	83.5	1 855 925	89.3		
3	200 806	85.8	1 965 384	90.2		
4	198 893	86.3	2 368 572	89.9	116 904	99.8
5 (highest)	228 499	86.3	3 011 373	90.2	109 890	99.7
Missing information	84 432	84.4	40 021	84.2	1 876	99.7
Proportion of people (25–64) without a diploma^a^						
1 (lowest)	208 167	88.9	2 019 781	92.3	127 329	99.8
2	213 978	86.8	2 641 612	91.1	111 476	99.8
3	184 839	85.8	2 264 162	89.6	97 545	99.8
4	166 385	81.6	2 095 298	88.4	80 671	99.8
5 (highest)	141 105	78.7	1 825 496	86.0	67 004	99.8
Missing information	84 432	84.4	40 199	84.2	1 876	99.7
Immigration Status^b^						
Immigrant (≤ 15 years ago)	93 253	81.2	1 037 992	90.7		
Non-immigrant (> 15 years ago)	905 653	85.1	9 848 556	89.4		

^a^Quintiles were derived from the Canadian Index of Multiple Deprivation by ranking postal codes and dividing them into five groups. For education, 1 = lowest and 5 = highest proportion without a high school diploma (least to most deprived). For visible minority, 1 = lowest and 5 = highest proportion of residents identifying as a visible minority

^b^Immigration status data was not available for Newfoundland

**Fig. 1:**
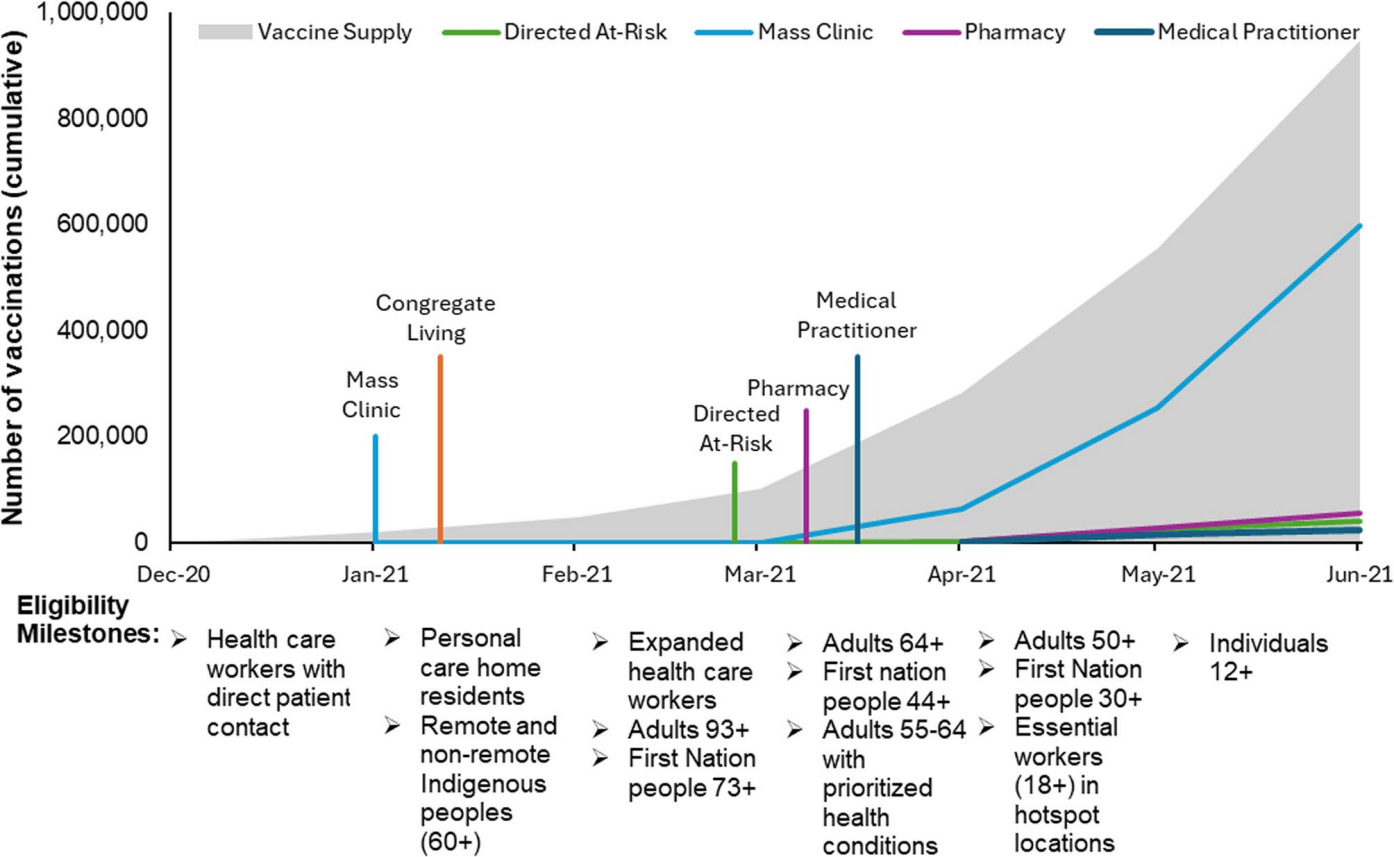
Timeline of vaccine rollout, eligibility, vaccine supply, and cumulative first doses by channel in Manitoba (Dec 2020-Jun 2021). [78] The vertical colored lines indicate the timing of the channel introduction; the height of these lines does not represent the number of vaccinations provided. The “Eligibility Milestones” indicate when additional groups became eligible for vaccination. These are shown along the timeline of the cumulative vaccinations by channel

**Fig. 2:**
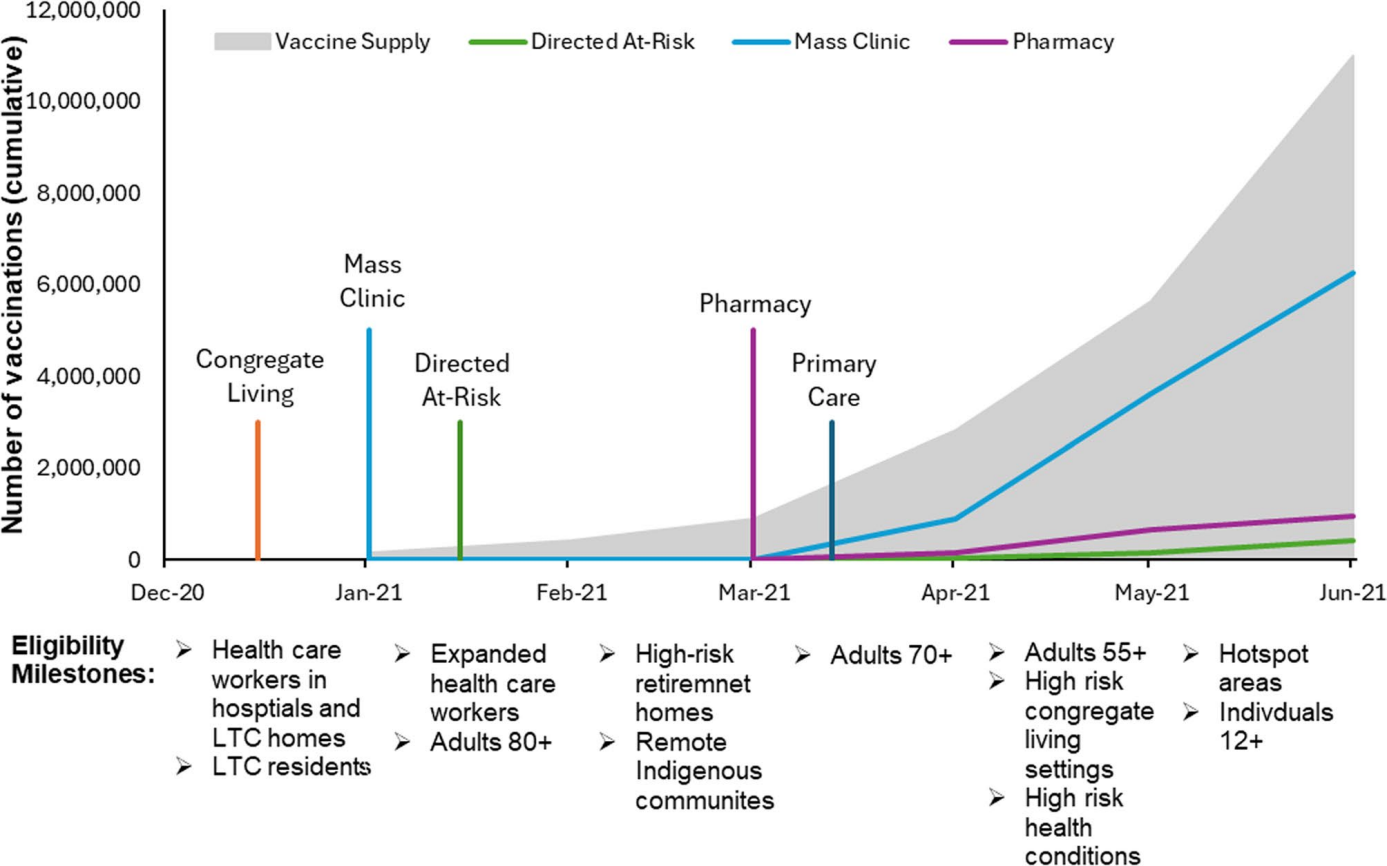
Timeline of vaccine rollout, eligibility, vaccine supply, and cumulative first doses by channel in Ontario (Dec 2020-June 2021). [78] The vertical colored lines indicate the timing of the channel introduction; the height of these lines does not represent the number of vaccinations provided. The “Eligibility Milestones” indicate when additional groups became eligible for vaccination. These are shown along the timeline of the cumulative vaccinations by channel

**Fig. 3:**
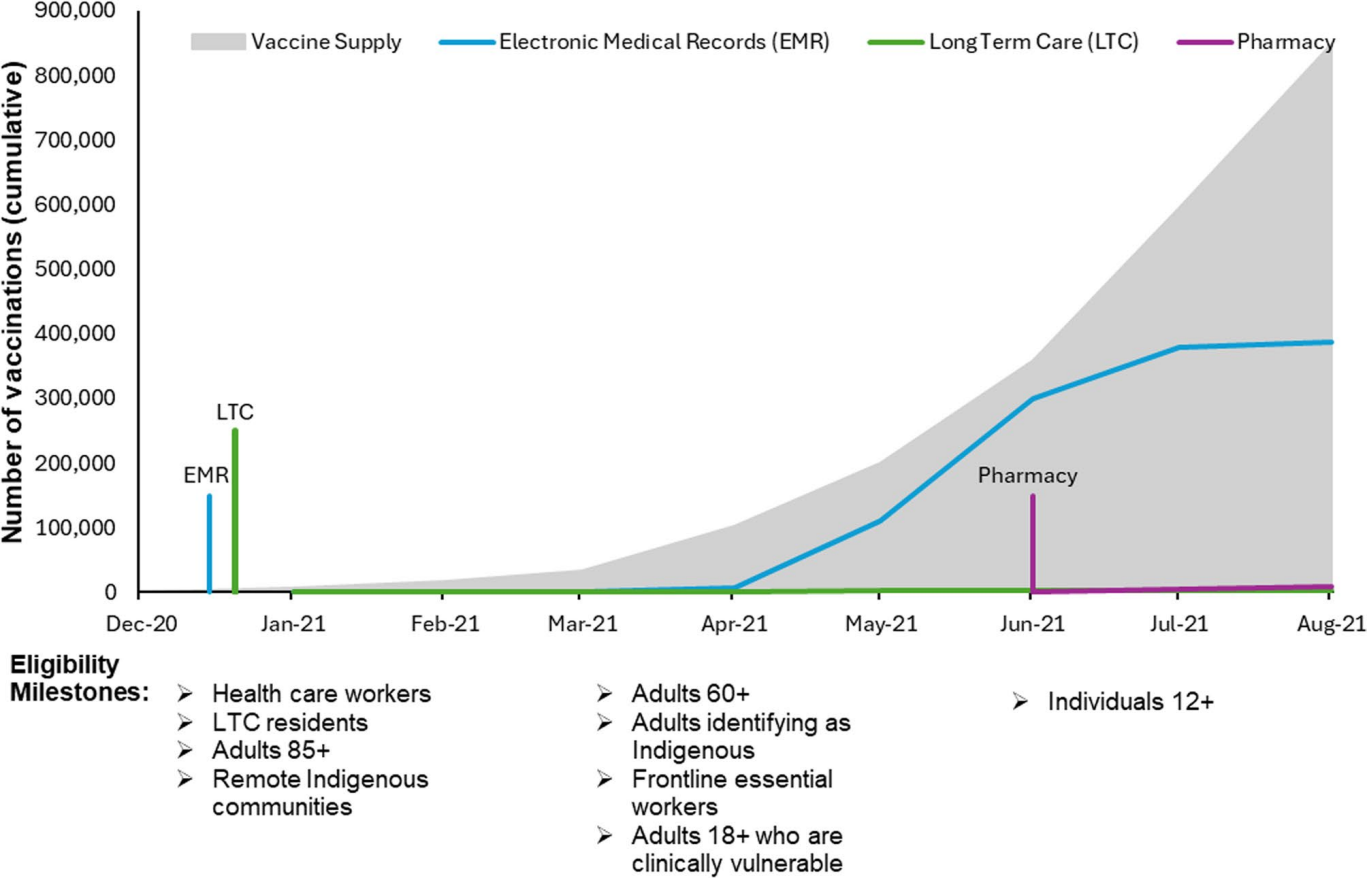
Timeline of vaccine rollout, eligibility, vaccine supply, and cumulative first doses by channel in Newfoundland (Dec 2020-Aug 2021). [78] The vertical colored lines indicate the timing of the channel introduction; the height of these lines does not represent the number of vaccinations provided. The “Eligibility Milestones” indicate when additional groups became eligible for vaccination. These are shown along the timeline of the cumulative vaccinations by channel

The timelines with vaccination eligibility, channel introduction, vaccine supply, and cumulative vaccinations provided for each province are presented below (Figs. 1, 2 and 3). While the mainstream major clinic categories (mass vaccination clinics in Ontario and Manitoba and EMR in Newfoundland) were introduced as the first channel in January, vaccine availability resulted in true mass vaccination being delayed till mid-February. The striking finding is the dramatic difference between the number of vaccines delivered via the mainstream major clinics and the other intentional equity focused channels, which barely appear on the graphs.

The analysis for each equity deserving group is presented in Tables 5, 6, 7 and 8 below. The data presented represents the available data from each jurisdiction. For example, while congregate living facilities in Ontario provided vaccinations, the data available did not facilitate including the introduction of this channel separately. Each table presents the channels where data was available, the total pre and post channel introduction vaccination rates for the equity-deserving group, and the change in the equity gap between the period before and after the introduction of the channel.

Table 5 presents the vaccination rates and changes comparing the highest and lowest income groups based on postal code of residence [76]. In Ontario, health care workers and certain age groups were prioritized early in the campaign, which indirectly resulted in earlier vaccination for some higher-income groups. There was a dramatic increase in rates of vaccination in both income groups one month after the introduction of the directed at-risk and mass channels (0.1 to 6.4% before and after channel introduction for the highest income group and 0 to 7.3% for the lowest income group) mainly due to the latter channel being introduced at almost the same time but with much larger capacity. The pharmacy channel was introduced later when the vaccination rates were increasing very rapidly (6.4 to 38.0% before and after channel introduction for the highest income group and 7.3 to 45% for the lowest income group) before and after introduction of the channel. The income equity gap was reduced in Ontario with the introduction of all three channels.

In Manitoba most vaccines were delivered through the mass vaccination clinics. The equity gap increased from the month before the introduction of the pharmacy and medical practitioner channels due to increased vaccinations through the mass immunization channel. Vaccination rates for the higher income group were lower than the lowest income group in Manitoba prior to the introduction of the pharmacy channel (1.4% compared to 1.5%), but this was reversed post pharmacy channel introduction (48.6% compared 38.6%).

There are three channels with data for Newfoundland. The dominant mainstream channel is labelled EMR reflecting the multiple channels that are indistinguishable in the data. It was introduced almost simultaneously with the Long-Term Care (LTC) channel.

**Table 5:** Percentages and 95% confidence intervals of vaccinated individuals by income quintile, pre/post vaccine channel introduction

		Highest income (Q1)			Lowest income (Q5)			Change in equity gap	
Province	Vaccine Channel	One month before introduction	One month after introduction	Change	One month before introduction	One month after introduction	Change	Difference in Differences (Q5-Q1)	Effect on gap^b^
Ontario	Congregate Living (Dec 15, 2020)	n/a^a^	4.0	n/a	n/a	4.6	n/a	n/a	n/a
	Mass Clinic (Jan 1, 2021)	0.1	6.4	6.4 (6.2, 6.5)	0	7.3	7.3 (7.1, 7.5)	0.9 (0.7, 1.1)	↓
	Directed At-Risk (Jan 15, 2021)	0.1	6.4	6.4 (6.2, 6.5)	0	7.3	7.3 (7.1, 7.5)	0.9 (0.7, 1.1)	↓
	Pharmacy (Mar 1, 2021)	6.4	38.0	31.6 (31.4, 31.7)	7.3	45.0	37.6 (37.5, 37.8)	6.1 (5.9, 6.3)	↓
Manitoba	Mass Clinic (Jan 1, 2021)	n/a	16.5	n/a	n/a	26.4	n/a	n/a	n/a
	Directed At-Risk (Feb 25, 2021)	n/a	28.5	n/a	n/a	30.8	n/a	n/a	n/a
	Pharmacy (Mar 8, 2021)	1.4	48.6	47.2 (46.9, 47.6)	1.5	38.6	37.1 (36.9, 37.4)	−10.1 (−10.5, −9.7)	↑
	Medical Practitioner (Mar 16, 2021)	12.0	50.1	38.2 (37.6, 38.7)	11.8	40.6	28.8 (28.4, 29.2)	−9.3 (−10.0, −8.7)	↑
Newfoundland	EMR (Dec 15, 2021)	n/a	8.8	n/a	n/a	5.4	n/a	n/a	n/a
	Long Term Care (Dec 20, 2021)	n/a	8.8	n/a	n/a	5.4	n/a	n/a	n/a
	Pharmacy (June 2021)	75.1	86.1	11.0 (10.4, 11.6)	64.1	75.9	11.9 (11.3, 12.4)	0.9 (0.1, 1.7)	↓

^a^N/A: no data on vaccination rates prior to the introduction of the channel resulting in no change in equity gap being available

^b^An upward pointing arrow indicates an increase in the vaccination gap between the highest and lowest income quintile and a downward pointing arrow indicates a decrease in the gap

Tables 6, 7 and 8 indicate that the equity gap increased with the introduction of the smaller channels for the immigrant (Table 6), visible minority (Table 7) and lower education (Table 8) populations, due to the rapid increase in vaccinations provided in the mainstream channels. Immigration data was not available in Newfoundland (Table 6).

**Table 6:** Percentages and 95% confidence intervals of vaccinated individuals by immigration status, pre/post vaccine channel introduction

		Non-immigrant (> 15 years) (Q1)			Immigrant (≤ 15 years) (Q5)			Change in equity gap	
Province	Vaccine Channel	One month before introduction	One month after introduction	Change	One month before introduction	One month after introduction	Change	Difference in Differences (Q5-Q1)	Effect on gap^b^
Ontario	Congregate Living (Dec 15, 2020)	n/a^a^	4.8	n/a	n/a	0.8	n/a	n/a	n/a
	Mass Clinic (Jan 1, 2021)	0.1	8.9	8.8 (8.8, 8.9)	0	1.5	1.5 (1.3, 1.7)	−7.3 (−7.5, −7.1)	↑
	Directed At-Risk (Jan 15, 2021)	0.1	8.9	8.8 (8.8, 8.9)	0	1.5	1.5 (1.3, 1.7)	−7.3 (−7.5, −7.1)	↑
	Pharmacy (Mar 1, 2021)	8.9	44.4	35.5 (35.4, 35.5)	1.5	25.2	23.7 (23.4, 23.9)	−11.8 (−12.0, −11.6)	↑
Manitoba	Mass Clinic (Jan 1, 2021)	n/a	36.3	n/a	n/a	0	n/a	n/a	n/a
	Directed At-Risk (Feb 25, 2021)	n/a	30.8	n/a	n/a	6.7	n/a	n/a	n/a
	Pharmacy (Mar 8, 2021)	2.2	45.8	43.6 (43.5, 43.8)	0	23.7	23.7 (23.2, 24.1)	−19.9 (−20.4, −19.5)	↑
	Medical Practitioner (Mar 16, 2021)	14.5	47.9	33.4 (33.2, 33.6)	0.4	23.7	23.3 (22.9, 23.8)	−10.1 (−10.6, −9.6)	↑

^a^N/A: no data on vaccination rates prior to the introduction of the channel resulting in no change in equity gap being available

^b^An upward pointing arrow indicates an increase in the vaccination gap between the non-immigrant population (born in Canada or immigrated more than 15 years ago) and the immigrant population (immigrated to Canada in the last 15 years) and a downward pointing arrow indicates a decrease in the gap

**Table 7:** Percentages and 95% confidence intervals of vaccinated individuals by visible minority quintile, pre/post vaccine channel introduction

		Low proportion of adults who self-identify as visible minority (Q1)			High proportion of adults who self-identify as visible minority (Q5)			Change in equity gap	
Province	Vaccine Channel	One month before introduction	One month after introduction	Change	One month before introduction	One month after introduction	Change	Difference in Differences (Q5-Q1)	Effect on gap^b^
Ontario	Congregate Living (Dec 15, 2020)	n/a^a^	2.5	n/a	n/a	5.2	n/a	n/a	n/a
	Mass Clinic (Jan 1, 2021)	0	7.3	7.3 (7.1, 7.5)	0.1	6.4	6.4 (6.2, 6.5)	−0.9 (−1.1, −0.7)	↑
	Directed At-Risk (Jan 15, 2021)	0	7.3	7.3 (7.1, 7.5)	0.1	6.4	6.4 (6.2, 6.5)	−0.9 (−1.1, −0.7)	↑
	Pharmacy (Mar 1, 2021)	7.3	45.0	37.6 (37.5, 37.8)	6.4	38.0	31.6 (31.4, 31.7)	−6.1 (−6.3, −5.9)	↑
Manitoba	Mass Clinic (Jan 1, 2021)	n/a	26.4	n/a	n/a	16.5	n/a	n/a	n/a
	Directed At-Risk (Feb 25, 2021)	n/a	30.8	n/a	n/a	28.5	n/a	n/a	n/a
	Pharmacy (Mar 8, 2021)	1.7	50.0	48.3 (47.9, 48.6)	1.2	35.3	34.1 (33.8, 34.4)	−14.2 (−14.6, −13.7)	↑
	Medical Practitioner (Mar 16, 2021)	12.6	52.3	39.7 (39.2, 40.2)	9.1	36.3	27.3 (26.9, 27.7)	−12.4 (−13.1, −11.7)	↑
Newfoundland	EMR (Dec 15, 2021)	n/a	6.3	n/a	n/a	9.1	n/a	n/a	n/a
	Long Term Care (Dec 20, 2021)	n/a	6.3	n/a	n/a	9.1	n/a	n/a	n/a
	Pharmacy (June 2021)	66.7	81.4	14.7 (14.3, 15.0)	71.8	81.7	9.9 (9.4, 10.5)	−4.8 (−5.4, −4.1)	↑

^a^N/A: no data on vaccination rates prior to the introduction of the channel resulting in no change in equity gap being available

^b^An upward pointing arrow indicates an increase in the vaccination gap between the quintile with the lowest proportion of adults who self-identify visible minority and the quintile with the highest proportion of adults who self-identify visible minority, and a downward pointing arrow indicates a decrease in the gap

**Table 8:** Percentages and 95% confidence intervals of vaccinated individuals by education level, pre/post vaccine channel introduction

		Low proportion of adults without a high school diploma (Q1)			High proportion of adults without a high school diploma (Q5)			Change in Equity Gap	
Province	Vaccine Channel	One month before introduction	One month after introduction	Change	One month before introduction	One month after introduction	Change	Difference in Differences (Q5-Q1)	Effect on gap^b^
Ontario	Congregate Living (Dec 15, 2020)	n/a^a^	5.6	n/a	n/a	3.0	n/a	n/a	n/a
	Mass Clinic (Jan 1, 2021)	0.2	10.0	9.8 (9.6, 10)	0	6.6	6.5 (6.4, 6.7)	−3.3 (−3.5, −3.0)	↑
	Directed At-Risk (Jan 15, 2021)	0.2	10.0	9.8 (9.6, 10)	0	6.6	6.5 (6.4, 6.7)	−3.3 (−3.5, −3.0)	↑
	Pharmacy (Mar 1, 2021)	10.0	47.8	37.9 (37.7, 38.1)	6.6	37.7	31.2 (31.0, 31.3)	−6.7 (−6.9, −6.4)	↑
Manitoba	Mass Clinic (Jan 1, 2021)	n/a	45.7	n/a	n/a	22.3	n/a	n/a	n/a
	Directed At-Risk (Feb 25, 2021)	n/a	30.7	n/a	n/a	24.2	n/a	n/a	n/a
	Pharmacy (Mar 8, 2021)	4.3	48.9	44.6 (44.2, 45.0)	0.7	40.1	39.5 (39.2, 39.8)	−5.1 (−5.6, −4.6)	↑
	Medical Practitioner (Mar 16, 2021)	21.9	52.0	30.1 (29.5, 30.7)	6.2	41.2	35.0 (34.6, 35.4)	4.9 (4.2, 5.6)	↓
Newfoundland	EMR (Dec 15, 2021)	n/a	8.5	n/a	n/a	4.4	n/a	n/a	n/a
	Long Term Care (Dec 20, 2021)	n/a	8.5	n/a	n/a	4.4	n/a	n/a	n/a
	Pharmacy (June 1, 2021)	66.7	81.4	14.7 (14.3, 15.0)	71.8	81.7	9.9 (9.4, 10.5)	−4.8 (−5.4, −4.1)	↑

^a^N/A: no data on vaccination rates prior to the introduction of the channel resulting in no change in equity gap being available

^b^An upward pointing arrow indicates an increase in the vaccination gap between the quintile with the lowest proportion of adults (25–64) without a high school diploma and the quintile with the highest proportion of adults without a high school diploma and a downward pointing arrow indicates a decrease in the gap

## Discussion

This study examined the impact of various vaccine delivery channels on vaccination rates among equity-deserving individuals. The natural experiment created by different jurisdictional approaches provided an opportunity to compare the effectiveness of diverse vaccine distribution strategies. Our findings indicate that equity gaps typically widened following the introduction of new vaccine distribution channels. Specifically, COVID-19 vaccination rates one month after implementing a new distribution channel increased less for individuals living in areas with higher proportions of self-identified visible minority adults compared to the general population. Similar disparities were observed for immigrants relative to those residing in Canada for more than fifteen years, and for individuals without a high school diploma compared to the general population. Notably, the gap decreased in Ontario and Newfoundland for those in the lowest income quintile, but this pattern was not replicated in Manitoba.

The profound impact of the COVID-19 pandemic precipitated an unprecedented mobilization of resources to develop vaccines to mitigate SARS-CoV-2-associated morbidity and mortality. The timelines demonstrate that each province responded distinctively to the challenge of implementing population-based vaccination programs with limited vaccine supply (Figs. 1, 2 and 3). All jurisdictions attempted to prioritize individuals at elevated risk of adverse outcomes from SARS-CoV-2 infection, based on available evidence [22]. These prioritization strategies, while necessary, also shaped early access and may have inadvertently delayed vaccine availability for some equity-deserving populations. Our results further suggest that vaccination rates increased as vaccine supply and distribution networks stabilized over time; however, access remained limited for certain populations. This is consistent with prior research, which shows that despite targeted distribution strategies aimed at reducing inequalities, gaps in vaccine coverage persisted through the vaccine rollout, including those related to age, educational attainment, household income, and race/ethnicity [13, 14]. 

 The urgency to increase population vaccination rates, coupled with the recognition of inequitable access for specific Canadian populations, led to public health initiatives implementing targeted vaccine distribution channels to enhance vaccination rates in hard-to-reach populations [13, 14]. For instance, the disproportionate impact of the 2009 H1N1 influenza outbreak on First Nations in Manitoba resulted in early vaccine provision to these communities [79]. 

Several factors may explain our findings. Examining outcomes merely one month after introducing new channels potentially overlooks the effects of established channels with greater vaccination capacity. Our data suggest that smaller, harder-to-reach channels received comparatively less vaccine supply and required more time for deployment. The number of vaccines provided through the equity-focused channels may have been masked by the simultaneous growth in vaccine provision through the mainstream clinics as eligibility increased amongst the general population. Furthermore, effective engagement with historically marginalized groups, such as visible minorities and those affected by multiple intersecting health determinants, necessitates community relationships and trust-building [13, 80, 81]. Consequently, vaccine channels intended for harder-to-reach groups may have been underutilized during relationship development phases. It is also important to consider that widening equity gaps may reflect broader structural limitations, such as the timing of rollout, logistical constraints, or prioritization policies, rather than the ineffectiveness of the delivery channels themselves. These findings may reflect the complex interplay of policy, capacity, and access barriers that shaped early implementation efforts. This study is part of a broader research study that examines these issues in greater depth. Notably, the qualitative findings from that work do not always align with our results [22]. These qualitative findings will be published in a forthcoming paper.

While access to provincial-level population-based vaccination data represents a significant strength of this study, several limitations warrant consideration. The three participating provinces employ different data structures and variable definitions, constraining interjurisdictional comparison. Newfoundland lacks access to linked immigration data. Income, educational achievement, high school graduation, and visible minority status were ecological variables based on postal codes stratified into quintiles [62]. While data are available for individuals, the data reflecting the channels were combined for some channels due to concerns about the validity of specific channel data. This methodological constraint obscured the impact of channels specifically designed to address equity-deserving populations behind larger mainstream channels, thereby preventing a definitive evaluation of targeted channels. Additionally, the use of a broad “visible minority’ category overlooks considerable diversity within this group. Not all subgroups experience the same systemic barriers or would be considered equity-deserving, highlighting the need for more disaggregated analyses in future research.

Despite these limitations, our analyses demonstrate the implementation of flexible, creative approaches to rapid population-wide vaccination efforts across all three jurisdictions. Data limitations precluded determination of whether specific channels contributed to narrowing vaccination rate disparities for specific equity-deserving populations. The various channels may have offered advantages such as proximity to residence or workplace, provision by trusted healthcare professionals, ease of scheduling, or reduced waiting times. The expanded options for vaccine access likely contributed to high population vaccination rates by providing multiple opportunities that addressed diverse barriers to vaccination, recognizing that individuals are more likely to pursue vaccination when their specific challenges are accommodated.

The data collection systems employed during the COVID-19 vaccination campaign proved inadequate for evaluating different vaccination approaches. Future public health emergency responses should prioritize uniform data collection across Canadian provinces to facilitate comprehensive evaluation of health system responses, including the capacity to monitor impacts on population inequities. Finally, efforts to develop functional learning health systems remain contingent upon robust data infrastructure to support their operation [82]. 

## Conclusion

The observed widening of equity gaps following the introduction of new vaccine distribution channels may stem from these channels initially offering vaccines at a smaller scale compared to mass vaccination clinics or it could reflect shortcomings in the existing data infrastructure. To address this, there is a pressing need for the standardized collection of data across Canada. A consistent, national approach to vaccine data collection and reporting is essential for evaluating the health system’s response in future pandemics, monitoring health inequities, and supporting coordinated public health decision-making. This will be especially important as Canada prepares for the rollout of A(H5) vaccines, where variations in strategy and uptake are again anticipated.

## Supplementary Information

Supplementary Material 1. Description of the databases used in the study

Supplementary Material 2. The RECORD reporting statement

Supplementary Material 3. Four multi-panel figures illustrating the cumulative monthly vaccination rates among eligible individuals in each province, broken down by the following equity-deserving groups: low-income, immigrant, visible minority, and individuals without a high school diploma

## Data Availability

The source data used in this study were originally collected during the routine administration of health and social services in Manitoba, Ontario, and Newfoundland and Labrador. Access was provided to the research team under data sharing agreements between the data trustees and the research institutions. The data are not owned by the researchers and cannot be deposited in a public repository. To review source data specific to this article or project, interested parties should contact the Alan Katz (corresponding author) at alan.katz@umanitoba.ca who will facilitate data access.
