# Supplementary material for: The impact of COVID-19 vaccine distribution channels on equity-deserving populations: a Canadian population-based cohort study using administrative data
Source: BMC Public Health. 2026 Jan 9;26:494. doi: 10.1186/s12889-025-24824-4 (PMC12882278; doi:10.1186/s12889-025-24824-4)
Supplement: Supplementary file 1 — Supplementary Material 1. Description of the databases used in the study [file 12889_2025_24824_MOESM1_ESM.docx]

**Table 1.** Description of databases used in the study analyses.

| **Database** | **Database Description** |
| --- | --- |
| **Manitoba** | |
| Manitoba Health Insurance Registry | A longitudinal population-based registry of demographic data for all individuals who have been registered with Manitoba Health at some point since 1970. |
| COVID-19 Vaccinations, appointments, and Screening Data | Data contains individual records for each COVID-19 vaccine recipient, including delivery location and provider type, as well as information about on-line vaccination appointments and patient screening. |
| COVID-19 Lab Testing and Results | Data on test information and results, acquisition information, patient age, postal code, and group membership indicator. |
| COVID-19 Surveillance Data | Data on infection and acquisition classifications, symptoms, case outcome, risk factors, exposure, interventions, and transmission assessments. |
| Canada Census | Contains social data based on population survey that include aggregate demographic information such as age, sex, marital status, employment, and income for all persons and housing units within each dissemination area in Canada. |
| Medical claims | Data includes claims for visits to physician/primary care providers in offices, hospital, and outpatient departments. |
| Hospital Abstracts | Data consists of hospital forms/computerized records containing summaries of demographic and clinical information completed at the point of discharge from hospital. |
| Immigration, Refugee, and Citizenship Canada Registry Data (IRCC) | A registry of immigrants to Canada who became permanent residents. |
| **Ontario** | |
| Ontario Drug Benefit Claims | Contains Ontario Drug Benefit information including recipients, payments, claims, and pharmacy and practitioner information. |
| Ontario Health Insurance Plan Claims Database | Contains data on all claim items processed including information such as patient, service, and amount paid. |
| Discharge Abstract Database | Contains administrative, clinical, and demographic information on hospital discharges (including deaths, sign-outs, and transfers). |
| National Ambulatory Care Reporting System | Contains demographic, administrative, clinical, and service-specific data for all hospital-based and community-based ambulatory care. |
| Registered Person Database | Contains core demographic information for persons registered under OHIP and those who are eligible for the Ontario Drug Program. |
| Continuing Care Reporting System | Contains demographic, clinical, functional, and resource utilization information on individuals who receive continuing care services in hospitals or long-term care homes in Canada. |
| IRCC Permanent Residents Database | A registry of immigrants to Canada who became permanent residents. |
| **Newfoundland and Labrador** | |
| NL Medical Care Plan (MCP) Beneficiary Registry | Contains demographic and administrative information on all Newfoundland and Labrador residents, landed immigrants, and some international workers and post-secondary students. |
| Provincial Drug Information System | Contains person-specific medication profiles including COVID-19 vaccination status and channel information for vaccines administered in pharmacies. |
| Meditech System | Contains electronic patient record, including COVID-19 vaccination status, in the acute care and long-term care systems. |
| Provincial Electronic Medical Record Program | Contains demographic and administrative information that enables the identification of individuals in the EHR. |
| 2016 Canada Census Data | Contains social data based on population survey that include aggregate demographic information such as age, sex, marital status, employment, and income for all persons and housing units within each dissemination area in Canada. |
