# Supplementary material for: The impact of COVID-19 vaccine distribution channels on equity-deserving populations: a Canadian population-based cohort study using administrative data
Source: BMC Public Health. 2026 Jan 9;26:494. doi: 10.1186/s12889-025-24824-4 (PMC12882278; doi:10.1186/s12889-025-24824-4)
Supplement: Supplementary file 3 — Supplementary Material 3. Four multi-panel figures illustrating the cumulative monthly vaccination rates among eligible individuals in each province, broken down by the following equity-deserving groups: low-income, immigrant, visible minority, and individuals without a high school diploma [file 12889_2025_24824_MOESM3_ESM.docx]

**
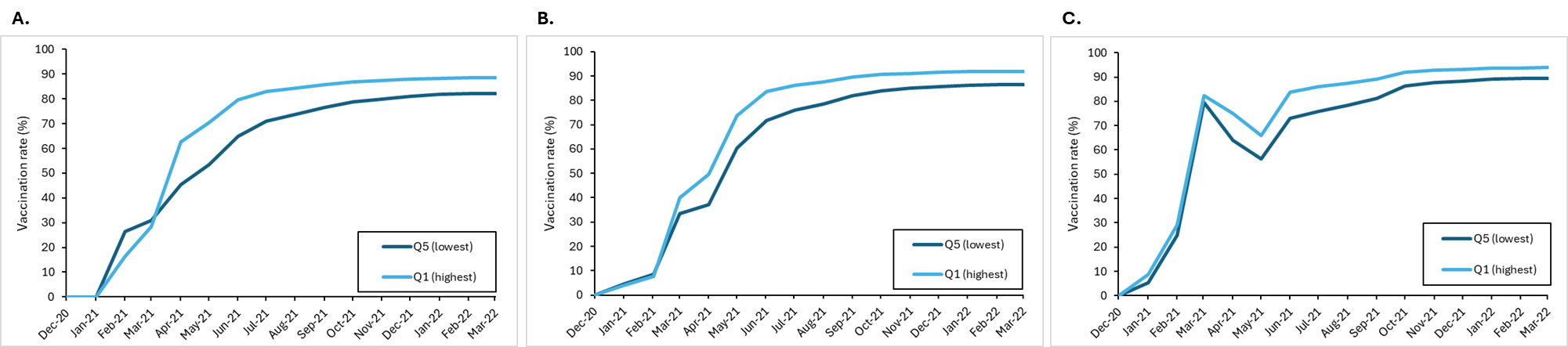
**

**Figure S1. Cumulative monthly vaccination rate of eligible individuals in (A) Manitoba, (B) Ontario, and (C) Newfoundland by income quintile (Dec 2020-Mar 2022).** Q5 is the lowest income quintile and Q1 is the highest income quintile. Rates were calculated using the eligible population in each month as the denominator; as eligibility expanded over time, this may result in apparent dips in the cumulative curves.

**
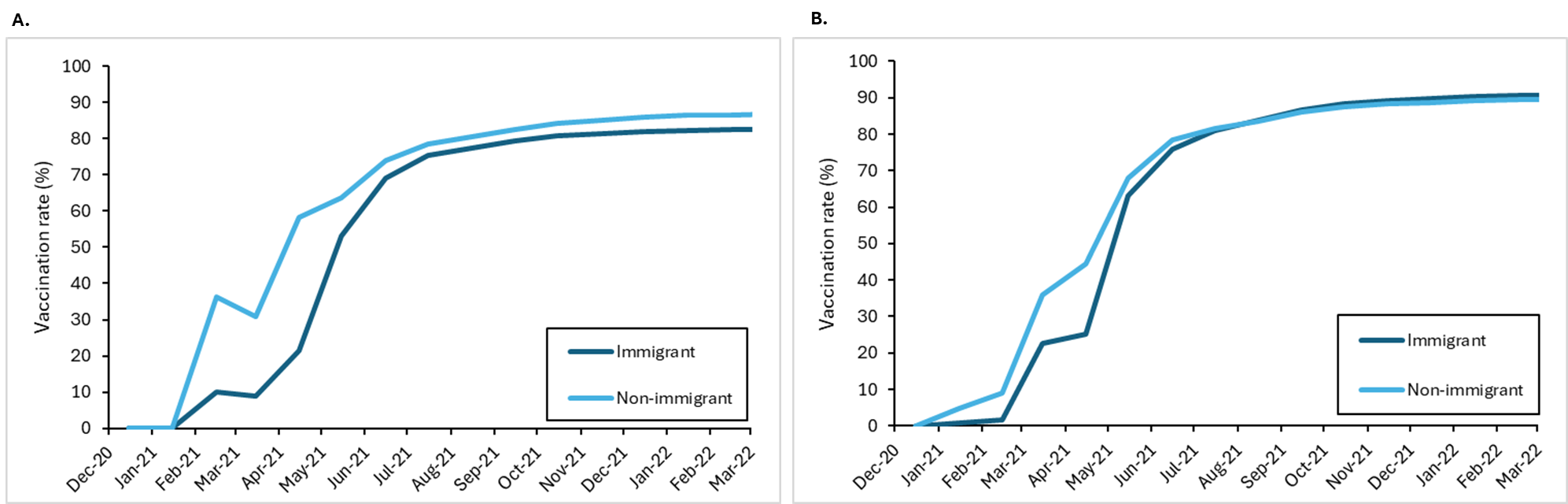
**

**Figure S2. Cumulative monthly vaccination rate of eligible individuals in (A) Manitoba and (B) Ontario by immigration status (Dec 2020-Mar 2022).** Anyone who was born in Canada or immigrated more than 15 years ago was classified as an non-immigrant. Anyone who immigrated to Canada in the last 15 years was considered an immigrant. Rates were calculated using the eligible population in each month as the denominator; as eligibility expanded over time, this may result in apparent dips in the cumulative curves.


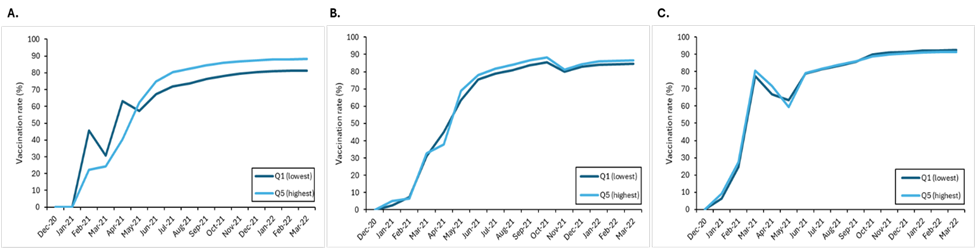


**Figure S3. Cumulative monthly population vaccination rate by proportion of visible minorities in (A) Manitoba, (B) Ontario, and (C) Newfoundland (Dec 2020-Mar 2022).** Q5 is the quintile with the highest proportion of individuals identifying as a visible minority. Rates were calculated using the eligible population in each month as the denominator; as eligibility expanded over time, this may result in apparent dips in the cumulative curves.

**
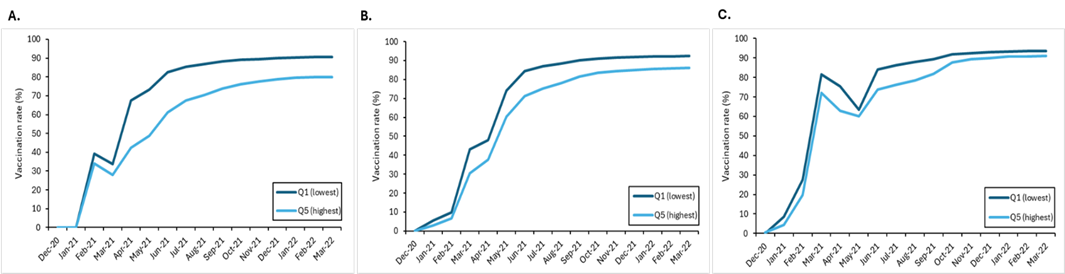
**

**Figure S4. Cumulative monthly population vaccination rate by education achievement level in (A) Manitoba, (B) Ontario, and (C) Newfoundland by education level (Dec 2020-Mar 2022).** Q5 is the highest proportion of adults without a high school diploma. Rates were calculated using the eligible population in each month as the denominator; as eligibility expanded over time, this may result in apparent dips in the cumulative curves.
